# Extracellular Proteins: Novel Key Components of Metal Resistance in Cyanobacteria?

**DOI:** 10.3389/fmicb.2016.00878

**Published:** 2016-06-07

**Authors:** Joaquín Giner-Lamia, Sara B. Pereira, Miquel Bovea-Marco, Matthias E. Futschik, Paula Tamagnini, Paulo Oliveira

**Affiliations:** ^1^Systems Biology and Bioinformatics Laboratory, Centro de Ciências do Mar, Universidade do AlgarveFaro, Portugal; ^2^Center for Biomedical Research, Universidade do AlgarveFaro, Portugal; ^3^Instituto de Investigação e Inovação em Saúde, Universidade do PortoPorto, Portugal; ^4^Instituto de Biologia Molecular e Celular, Universidade do PortoPorto, Portugal; ^5^Faculdade de Ciências, Departamento de Biologia, Universidade do PortoPorto, Portugal

**Keywords:** metal resistance, cyanobacteria, exoproteome, secretion, biotechnology

## Abstract

Metals are essential for all living organisms and required for fundamental biochemical processes. However, when in excess, metals can turn into highly-toxic agents able to disrupt cell membranes, alter enzymatic activities, and damage DNA. Metal concentrations are therefore tightly controlled inside cells, particularly in cyanobacteria. Cyanobacteria are ecologically relevant prokaryotes that perform oxygenic photosynthesis and can be found in many different marine and freshwater ecosystems, including environments contaminated with heavy metals. As their photosynthetic machinery imposes high demands for metals, homeostasis of these micronutrients has been widely studied in cyanobacteria. So far, most studies have focused on how cells are capable of controlling their internal metal pools, with a strong bias toward the analysis of intracellular processes. Ultrastructure, modulation of physiology, dynamic changes in transcription and protein levels have been studied, but what takes place in the extracellular environment when cells are exposed to an unbalanced metal availability remains largely unknown. The interest in studying the subset of proteins present in the extracellular space has only recently begun and the identification and functional analysis of the cyanobacterial exoproteomes are just emerging. Remarkably, metal-related proteins such as the copper-chaperone CopM or the iron-binding protein FutA2 have already been identified outside the cell. With this perspective, we aim to raise the awareness that metal-resistance mechanisms are not yet fully known and hope to motivate future studies assessing the role of extracellular proteins on bacterial metal homeostasis, with a special focus on cyanobacteria.

## Introduction

In comparison to other prokaryotes, the photosynthetic machinery in cyanobacteria imposes higher demand for metals (Cavet et al., 2003; Shcolnick and Keren, 2006; Huertas et al., 2014). Iron (Fe) is necessary for all three photosynthetic electron transfer chain complexes, manganese (Mn) for the water-splitting complex, copper (Cu) for plastocyanin and cytochrome *c* oxidase, magnesium (Mg) for chlorophylls, and zinc (Zn) for carbonic anhydrase (Cavet et al., 2003; Shcolnick and Keren, 2006; Castielli et al., 2009; Huertas et al., 2014). Thus, cyanobacteria are excellent model organisms to unravel the mechanisms for maintaining physiologically compatible intracellular metal concentrations. For their study, different approaches have been used, including targeted mutagenesis, random knockout libraries, and DNA microarrays. In addition, proteomic tools have been applied to obtain a holistic perspective of the cyanobacterial metal adaptive mechanisms. The vast majority of studies have focused on intracellular mechanisms underlying metal homeostasis (henceforth MH). Recently, several findings have indicated that extracellular proteins might play key roles in cyanobacterial metal resistance (henceforth MR), despite little being known of their response to stresses posed by metal availability. Here, after presenting a brief overview of cyanobacterial proteomic adaptations to conditions of unbalanced metal concentrations, we highlight the renewed interest in studying the cyanobacterial exoproteome. We will focus on two well studied metal-related cyanobacterial proteins that have been identified in the extracellular space, and will present novel results implicating the function of a TolC-homolog in metal-resistance mechanisms in cyanobacteria. We conclude by suggesting approaches that can address the salient question of how far MR in cyanobacteria is dependent on the function of extracellular proteins. Finally, we would like to remind the reader that this is a perspective article focusing on the potential role of extracellular proteins in MR. For more comprehensive overviews on the numerous mechanisms involved in MH in cyanobacteria we refer to recent reviews (Huertas et al., 2014; Cassier-Chauvat and Chauvat, 2015).

## Cyanobacterial Proteomic Responses to Unbalanced Metal Availability

A common response when cyanobacteria are stressed by metal availability is a change in the levels of proteins involved in central metabolic processes, including photosynthesis and carbon metabolism (e.g., Bhargava et al., 2008; Prakash et al., 2011; Cox and Saito, 2013; Mehta et al., 2014; Mota et al., 2015a,b). Studies performed with *Synechocystis* sp. PCC6803 (henceforth *Synechocystis*) exposed to nickel (Ni), cobalt (Co), or cadmium (Cd) showed an overall decrease in the levels of proteins related to photosynthesis or carbon metabolism or both (Bhargava et al., 2008; Mehta et al., 2014). A similar decrease of protein abundance was observed in *Anabaena* sp. PCC7120 (henceforth *Anabaena*) after Fe starvation (Prakash et al., 2011), and in *Cyanothece* sp. CCY0110 cultivated in media supplemented with Cu or Cd (Mota et al., 2015a,b). These and other results suggest an adaptive response of photosynthetic activity, and reorganization of carbon fluxes in response to physiological inadequate metal concentrations (Mota et al., 2015a,b). Given that metals are essential for the correct folding and functioning of many enzymes and can undergo undesirable redox reactions (Waldron and Robinson, 2009; Huertas et al., 2014), chaperones and anti-oxidative stress-related mechanisms are often activated after metal stress. For example, the levels and activity of superoxide dismutase were shown to increase upon metal exposure in different strains (Bhargava et al., 2008; Prakash et al., 2011; Mota et al., 2015a,b). Interestingly, although metal stress affects levels of chaperones in general, different chaperones respond differently to different metal concentrations, some being specific to a given metal, such as the heat-shock protein HspA whose levels increase upon exposure to Ni (Huertas et al., 2014; Mehta et al., 2014). Exposure to Ni also leads to an increase in the abundance of the cation efflux system protein NrsB, which is involved in Ni and Co tolerance (García-Domínguez et al., 2000; Mehta et al., 2014), suggesting that expression of specific membrane efflux complexes represents another level of cellular response under such stressful conditions. Recent studies also highlight the interaction between the metabolism of different metals as well as of metals with other nutrients (Cox and Saito, 2013; Mehta et al., 2014). Interestingly, in marine cyanobacteria, which dwell in oligotrophic environments, “genomic islands” have been described, representing highly variable regions predicted to encode proteins involved in niche adaptation (Coleman et al., 2006). In a coastal marine *Synechococcus* strain, such proteins have been shown to confer tolerance to Cu (Stuart et al., 2013), supporting their adaptive advantage as well as suggesting a mechanism driving population structure in environmental settings, in this case in response to metal availability.

## Cyanobacterial Exoproteome

As illustrated by the proteomic studies discussed, the cyanobacterial proteomic profile and dynamics of intracellular processes have been investigated under conditions of metal stress. However, one “compartment” remains to be analysed with the same level of detail: the extracellular space. The recognition of the dynamic role played by extracellular proteins in development, cell wall biogenesis, adhesion, and biofilm formation in several bacteria (Caccia et al., 2013) has motivated significant efforts in identifying and characterizing the cyanobacterial exoproteome [defined as the subset of proteins present in the extracellular space, actively secreted or not (Desvaux et al., 2009)].

Morphologically different (e.g., Christie-Oleza et al., 2015a; Hahn et al., 2015), metabolically distinct cyanobacteria (e.g., Vilhauer et al., 2014; Oliveira et al., 2016), and cyanobacteria occupying different ecological niches (e.g., Gao et al., 2014; Christie-Oleza et al., 2015b; Stuart et al., 2016) have been studied, giving us a first overview of the type of extracellular proteins secreted by these organisms. Three findings emerging from these studies are striking: (i) The large number of exoproteins found, covering many functional categories; (ii) The high proportion of proteins annotated as hypothetical or with unknown function; and (iii) The variation of exoproteome composition when cells are cultivated in different growth conditions. The latter aspect is especially relevant as it suggests that cyanobacteria have the capacity of actively modulating their exoproteome, through regulation of protein secretion in response to environmental stimuli, similarly to the intracellular proteome. Hence, cyanobacteria might seek to restore the homeostatic balance when exposed to stressful conditions not only by adapting their intracellular proteomes to that particular condition but also by adjusting the proteomic composition of their extracellular milieu.

When analyzing the cyanobacterial exoproteomes profiles described so far, it is interesting to find that there is a high incidence of strain-specific proteins (Christie-Oleza et al., 2015a). These can be associated with mutualistic or hostile interactions, with defence mechanisms (Oliveira et al., 2015a) or reflect the organism’s life strategy (Christie-Oleza et al., 2015a; Oliveira et al., 2016). Nevertheless, proteins that are common across species can also be found (Christie-Oleza et al., 2015a; Oliveira et al., 2015a), including exoproteins with a probable role in mixotrophy.

A functional role of the exoproteome in metal acquisition and resistance is supported by the detection of putative metal-binding proteins in the extracellular space. These proteins include metallothioneins, metallothionein-related proteins, ferritin, the photosystem II manganese-stabilizing protein, plastocyanin, and proteins annotated simply as “metal-binding proteins” in marine *Synechococcus* (Christie-Oleza et al., 2015a); FutA2 in *Synechocystis* (Cheregi et al., 2015; Oliveira et al., 2016) and homologs in marine *Synechococcus* (Christie-Oleza et al., 2015a); CopM in *Synechocystis* (Giner-Lamia et al., 2015) and homolog All7633 in *Anabaena* (Oliveira et al., 2015a); and ferritin-like Dps proteins in *Anabaena* (Oliveira et al., 2015a). As the function of many exoproteins remains uncharacterized, it is likely that other metal-binding proteins may be found in the extracellular space. From the proteins highlighted above, most are characterized as having a role in intracellular functions [e.g., metallothioneins, which are small cysteine-rich proteins having the capacity to bind different metals via the thiol group of its cysteine amino-acids and to sequester surplus atoms of metal (Huckle et al., 1993)]. However, their role as exoproteins in the extracellular space remains unclear.

The general mechanisms for metal resistance and homeostasis in cyanobacteria have been recently reviewed (Huertas et al., 2014). Here, we will illustrate the potential importance of exoproteome in the context of CopM and FutA2, whose roles in MH mechanisms have been investigated in detail.

### CopM, a Novel Cu Chaperone Found in the Extracellular Space of *Synechocystis*

**FIGURE 1:**
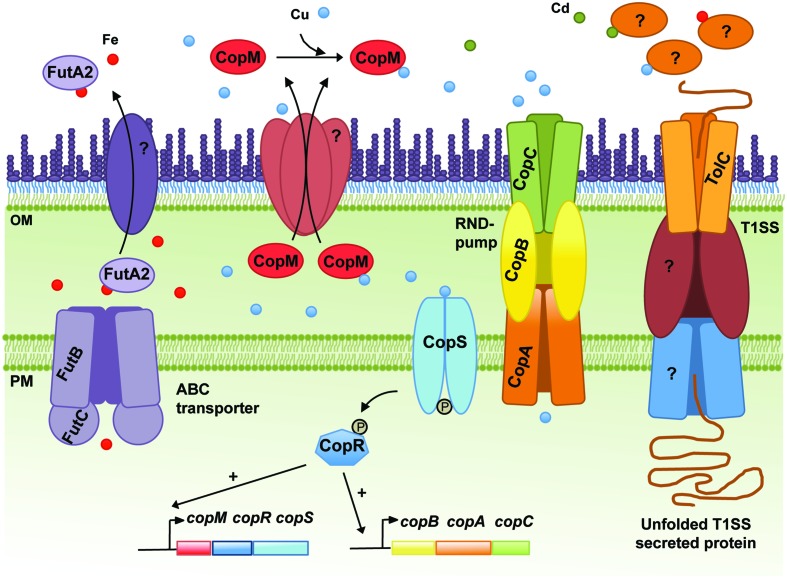
**Schematic representation of the metal homeostasis components in the cyanobacterium *Synechocystis* sp. PCC6803 discussed in the text.** The Resistance-Nodulation-Division (RND) pump CopBAC, the type I secretion system (T1SS), including TolC, and the ABC-type transporter dedicated to Fe import (FutBC) are also shown. Proteins mediating translocation of FutA2 and CopM from the periplasm to the extracellular space, and the remaining elements composing the T1SS are represented and marked with “?,” as their identities remain unknown. In addition, the molecular mechanisms triggering secretion of FutA2 to the extracellular space and any function there remain unclear. Potential exoproteins involved in metal resistance/homeostasis secreted by the T1SS or other protein secretion systems are depicted in the extracellular space. In the cytoplasm, CopRS-dependent activation of the *copMRS* and *copBAC* operons transcription is also illustrated. OM, outer membrane; PM, plasma membrane.

Copper resistance in *Synechocystis* comprises a two-component system, CopRS, a Cu-binding protein, CopM, and a Heavy Metal Efflux-Resistance-Nodulation-Division (RND) family export system, CopBAC (**Figure 1**) (Giner-Lamia et al., 2012). These proteins are encoded by two operons, *copMRS* and *copBAC*, regulated by CopRS in response to Cu in the medium. Mutants of *copRS* and *copBAC* genes are sensitive to Cu and also accumulate higher amounts of intracellular Cu than the wild-type. Recently, it was shown that CopM is able to bind one equivalent of Cu(I) or less tightly Cu(II), and that is found both in the periplasm and in the extracellular milieu of *Synechocystis* (Giner-Lamia et al., 2015). Deletion of *copM* renders cells as Cu-sensitive as those lacking *copB*, but less sensitive than a mutant lacking either *copR* or the *copRS* system, suggesting that both CopM and CopBAC constitute two independent resistance mechanisms in *Synechocystis*. Although CopM has been immuno-detected in the extracellular space, its role outside the cell is not yet known. Nevertheless, a role for CopM in preventing Cu accumulation has been suggested, probably by direct Cu-binding outside of the cell (Giner-Lamia et al., 2015) (**Figure 1**); alternatively, CopM could have an inhibitory role in Cu-import by an unknown mechanism. In either case, CopM could serve for Cu sequestration in the extracellular space, which is supported both by its Cu-binding capacity and its presence in such an environment.

CopM-like proteins have been identified in other cyanobacteria, but seem to be completely absent in *Prochlorococcus* strains and poorly represented in marine *Synechococcus* (see Supplementary Material). In *Anabaena*, All4988, All7594, and All7633 are CopM homologs (Giner-Lamia et al., 2015). In addition to a high degree of similarity between these proteins and CopM, their respective genes are located in the vicinity of Cu-related genes and their transcription has been shown to respond to Cu (Giner-Lamia et al., 2015). Furthermore, the product of the *all7633* gene was detected in the exoproteome of *Anabaena* (Oliveira et al., 2015a). All these data point to the possibility that, at least in cyanobacteria, CopM-like proteins could be a new family of proteins involved in Cu resistance in the extracellular environment. Interestingly, several functions have been assigned to functionally related protein families consisting of periplasmic Cu chaperones such us Cu buffering [PcoE (Zimmermann et al., 2012)], shuttling Cu to and from transporters [CusF or CopC (Djoko et al., 2007; Mealman et al., 2012)], or donating Cu to Cu-containing proteins [PrrC or Sco, Badrick et al., 2007; Blundell et al., 2013)]. However, a direct role in Cu-export or Cu-binding in the extracellular space has not been described yet. Future studies of CopM homologs and other periplasmic Cu chaperones across different bacterial groups will help to clarify whether their function can be extended to the extracellular milieu.

### FutA2, a Periplasmic Fe-Binding Protein with Unknown Function in the Extracellular Space of *Synechocystis*

In *Synechocystis*, the Fut Fe transporter is comprised of four proteins: two substrate-binding subunits (FutA1 and FutA2), a membrane associated subunit (FutB) and the ATP-binding component FutC (Katoh et al., 2001a). FutA1 and FutA2 are thought to act as ferric-binding proteins facilitating Fe(III) uptake. FutA2 has been identified in the periplasm (Fulda et al., 2000) and is the most abundant soluble Fe-binding protein in the periplasm of *Synechocystis* (Waldron et al., 2007), while FutA1 was detected neither in the soluble protein fraction of the periplasm nor in the cytoplasmic membrane (Tölle et al., 2002). These results, in addition to biochemical analyses suggest that FutA2 is the primary Fe-binding protein acting in Fe-uptake in *Synechocystis* (Badarau et al., 2008), while FutA1 is playing a role intracellularly, likely to protect photosystem II (Tölle et al., 2002). Nevertheless, Δ*futA1* and Δ*futA2* mutants present 63% and 16% reduction in Fe-uptake activity, respectively, compared to that of the wild-type (Katoh et al., 2001b). These observations seem contradictory, but the severely impaired Fe-uptake phenotype of Δ*futA1* could be explained by a coincident decline in FutA2 within the periplasm of this strain (Badarau et al., 2008). Periplasmic accumulation of FutA2 shows an intriguing dependence upon cytosolic FutA1 (Badarau et al., 2008) and highlights that regulation of the Fe-uptake system in *Synechocystis* is not entirely understood. To further illustrate this regulatory complexity, it should be noted that when *Synechocystis* cells were exposed to Cd, Ni, or Co, the levels of FutA2 decreased, while transcriptional analyses of *futA2* mRNA levels indicated that its transcription is indeed up-regulated in Cd and Co (Mehta et al., 2014). The authors interpreted these results as a possible post-translational regulation (Mehta et al., 2014). However, another hypothesis can be raised; in two recent studies, FutA2 was identified in the extracellular environment of *Synechocystis* cultivated under standard growth conditions (Cheregi et al., 2015; Oliveira et al., 2016), suggesting that FutA2 can be translocated from the periplasm to the extracellular space. Hence, under certain stressful conditions (as the ones described by Mehta et al., 2014), it is possible that FutA2 is further translocated to the extracellular space, explaining the lower FutA2 levels in the cells despite increased gene expression. Why FutA2 is displaced to the extracellular space is not clear. Additional studies are needed to understand what the role of FutA2 is in the extracellular space and how it relates to MR. Interestingly, FutA2-encoding genes are spread throughout the cyanobacterial phylum, being absent only in the Stigonematales (except in *Mastigocladopsis repens*, see Supplementary Material).

## Secretion Mechanisms for Cyanobacterial Proteins Await Elucidation

In addition to the challenge of identifying novel extracellular players involved in the mechanisms of MR, there is also a need to clarify which systems are used by bacteria to translocate proteins from the cytoplasm to the extracellular space. In the case of CopM, Western blot analysis showed that the protein has a signal peptide, which is cleaved off upon translocation from the cytoplasm to the periplasm (Giner-Lamia et al., 2015). This observation suggests that CopM is translocated to the extracellular space as a type II secretion system (T2SS) dependent substrate. Alternatively, FutA2 is a substrate of the Tat-dependent pathway (Waldron et al., 2007), and thus likely a T2SS-dependent substrate as well. Protein secretion mechanisms in cyanobacteria have remained poorly described. However, this is changing with two recent studies addressing the topic (Hahn et al., 2015; Oliveira et al., 2016). Despite being performed in different strains, both focused on the T1SS, which relies on the function of a tripartite complex composed of an outer-membrane protein (TolC), a cytoplasmic membrane ABC-type transporter, and a periplasmic counterpart. TolC is a homotrimeric protein that forms a channel-tunnel in the outer membrane (OM) and interacts with different plasma-membrane protein complexes, which confer substrate specificity (Delepelaire, 2004). In bacteria, TolC is implicated in a variety of diverse cellular functions mediating secretion of different substrates such as toxins, proteases and intracellular metabolites (Koronakis, 2003; Bleuel et al., 2005; Tatsumi and Wachi, 2008). As suggested by its versatile role, TolC-encoding genes are found in all cyanobacterial genomes analysed (see Supplementary Material). In *Synechocystis*, disruption of *tolC* (*slr1270*) was shown to impair protein secretion and to increase antibiotic sensitivity (Oliveira et al., 2016).

### TolC Could Be the Gateway for Secreting Metal Stress Related Proteins in *Synechocystis*

**FIGURE 2:**
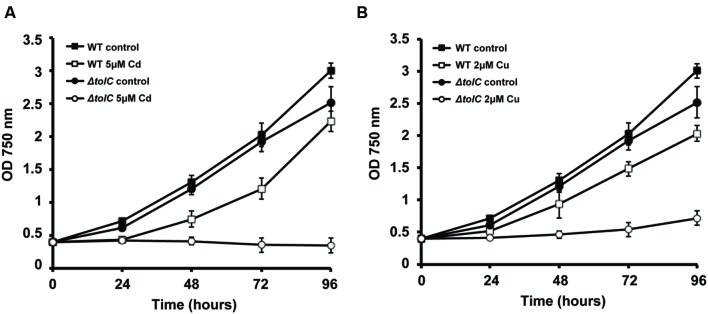
**Effect of Cu and Cd in the growth of *Synechocystis* sp. PCC6803 wild-type (WT) and *slr1270* (*tolC*) deletion-mutant.** Cells were grown in BG11C (Lopez-Maury et al., 2003) in Erlenmeyer flasks containing 5 μM of cadmium **(A)** or 2 μM of copper **(B)**, or without any metal supplementation, with a light intensity of 40 μE m^-2^ s^-1^. Growth was monitored following the OD_750_. Error bars correspond to standard deviation from at least three independent experiments.

In our laboratories, we continue to characterize the *slr1270*-deletion mutant under different conditions, aiming at better understanding the processes in which this TolC-homolog is involved. When *Synechocystis* wild-type and the *slr1270*-deletion mutant cells were cultivated in medium supplemented with selected metals, the mutant presented increased sensitivity to Cu and Cd (**Figure 2**), but not to Zn or Ni (data not shown). Interestingly, in *Salmonella enterica* serovar Typhimurium, TolC was also shown to contribute to Cu resistance, as a *tolC*-deletion mutant is more sensitive to the presence of Cu in the medium than the wild-type (Nishino et al., 2007). In addition, independent work on that same strain demonstrated that not only the *tolC* mutant has a reduced Cu tolerance, but it also accumulates Cu at non-lethal concentrations in the cytoplasm (Pointon, 2014). Altogether, these results point out that our understanding of Cu-resistance and transport mechanisms, and likely of other metals as well are not complete in bacteria, and that there is an involvement of TolC in the mechanisms of MR and transport across membranes.

A role of TolC is further supported given its homology to other outer-membrane proteins, including CusC, a component of the CusCFBA determinant of *E. coli* that mediates Cu-resistance (Franke et al., 2003). CusC was shown to be required *in vivo* for full Cu-resistance in *E. coli* (Franke et al., 2003). Interestingly, TolC has a similar architecture compared to that of CusC, and structural analyses suggest that the open channels in the respective assembled pumps may also be similar (Kulathila et al., 2011). Nevertheless, TolC does not restore metal resistance in a *cusC*-deletion mutant, suggesting that the three subunits of the putative CusCBA transenvelope efflux complex are all essential for *cus*-mediated Cu-resistance (Franke et al., 2003). The most likely reason for this observation would be an inability of TolC to form a functional pump with CusAB (Kulathila et al., 2011). Alternatively, TolC could have a potential role in metal chaperone secretion. However, as the TolC-dependent T1SS is described to translocate unfolded proteins (Delepelaire, 2004), TolC’s possible role suggested here can only be regarded in light of metal chaperones that bind the metal in the extracellular environment, and that through that binding “regulate” its availability for import. In the case of *Synechocystis*, TolC would transport proteins with similar properties to CopM and bind metals outside the cells, avoiding their deleterious side effects in the extracellular space or inside the cell. Either way, this result (**Figure 2**) highlights TolC and the exoproteome as new important elements to consider in MR in *Synechocystis*. Additional work needs to be carried out in order to clarify the exact role of TolC and the proteins present in the extracellular space in cyanobacteria, opening a new exciting field of research in MH.

## Outlook

Several aspects will have to be analyzed to fully understand how extracellular proteins may contribute to MR in cyanobacteria, including:

1. Dynamics of exoproteome: most of the cyanobacterial exoproteome studies available simply profile the exoproteins composition in a given growth condition, at a particular time point. More experiments will have to be performed to evaluate the dynamic exoproteome changes in response to specific environmental conditions. Particularly in terms of metals, this could be a valid approach to identify novel (extracellular) players involved in the mechanisms of MR. Furthermore, the technical limitations inherent to the analysis of the exoproteome may be overcome by enriching the sample in metal-binding proteins, by making use of immobilized metal-ion affinity chromatography.
2. Mechanisms of protein secretion: here we propose that TolC has a role in MR in *Synechocystis*, through export of components of the exoproteome, but this role needs to be confirmed and other protein secretion systems in cyanobacteria identified and characterized.
3. Indirect role of exoproteins in MR: a final aspect warranting further investigation is a potential interplay with extracellular polymeric substances (EPS) that also protect against deleterious metals and are involved in the sorption of essential ones (Kumar et al., 2007; Jittawuttipoka et al., 2013; Cassier-Chauvat and Chauvat, 2015). In fact, EPS structural exoproteins (Oliveira et al., 2015b) may also have an indirect role on MR by contributing to retain EPS metal-binding capacity.

Further progress in dissecting the composition and function of cyanobacterial exoproteomes will not only help to complete our understanding of MR in cyanobacteria, but might also be of biotechnological value. As cyanobacteria are being continuously engineered to become robust cell factories, breakthroughs in protein secretion and metal chaperone could increase their potential to become a valuable environmentally friendly tool for heavy-metal bioremediation.

## Author Contributions

JG-L and PO devised the outline of the perspective. JG-L designed the experiments, and MB-M and JG-L conducted the experimental work. MB-M, JG-L, and MEF analyzed the data. JG-L, SBP, and PO carried out literature search and wrote the manuscript. JG-L, SBP, MEF, PT, and PO contributed to discussion of content and review/editing before submission.

## Conflict of Interest Statement

The authors declare that the research was conducted in the absence of any commercial or financial relationships that could be construed as a potential conflict of interest.
