# Supplementary material for: Extracellular Proteins: Novel Key Components of Metal Resistance in Cyanobacteria?
Source: Front Microbiol. 2016 Jun 7;7:878. doi: 10.3389/fmicb.2016.00878 (PMC4894872; doi:10.3389/fmicb.2016.00878)
Supplement: Supplementary file 1 [file Presentation_1.PDF]

Supplementary Material

Extracellular proteins: Novel key components of metal homeostasis in cyanobacteria?

Joaquín Giner-Lamia*, Sara B. Pereira, Miquel Bovea-Marco, Matthias E. Futschik, Paula Tamagnini, Paulo Oliveira*

*** Correspondence:** Corresponding Authors: jglamia@sysbiolab.eu and paulo.oliveira@ibmc.up.pt

Supplementary Figure 1. Distribution of TolC (yellow box), CopM (green box) and FutA2 (blue box) within the cyanobacterial phylum. The number of proteins containing the typical domains in the theoretical proteome of each strain is indicated within the box. Empty boxes correspond to the absence of CopM and/or FutA2. The cyanobacterial phylogenetic tree was generated by the maximum likelihood analysis of 31 conserved proteins concatenated by Shih et al., 2013. The proteins TolC, CopM and FutA2 of *Synechocystis* sp. PCC6803 were screened for their Pfam domains using available tools in the Integrated Microbial Genomes (IMG) database (v4.530, June 2015 https://img.jgi.doe.gov/cgi-bin/m/main.cgi). The presence or absence of genes encoding proteins with the selected Pfam domains was investigated in 126 cyanobacterial genomes, also available at the IMG. The list of encoding genes was refined in tblastn searches using the *Synechocystis* sp. PCC6803 sequences as query.

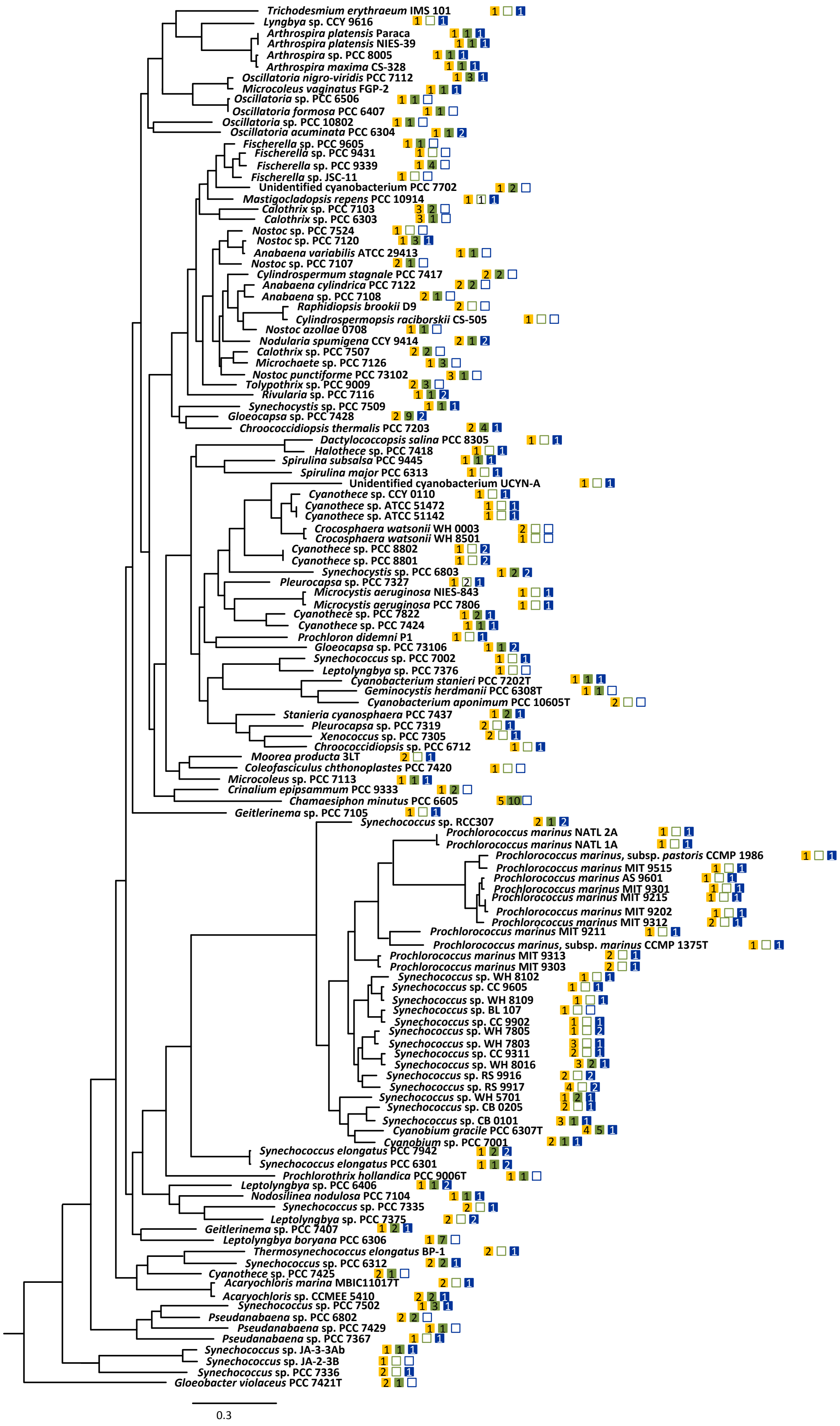
Trichodesmium erythraeum IMS 101
Lyngbya sp. CCY 9616
Arthrospira platensis Paraca
Arthrospira platensis NIES-39
Arthrospira sp. PCC 8005
Arthrospira maxima CS-328
Oscillatoria nigro-viridis PCC 7112
Microcoleus vaginatus FGP-2
Oscillatoria sp. PCC 6506
Oscillatoria formosa PCC 6407
Oscillatoria sp. PCC 10802
Oscillatoria acuminata PCC 6304
Fischerella sp. PCC 9605
Fischerella sp. PCC 9431
Fischerella sp. PCC 9339
Fischerella sp. JSC-11
Unidentified cyanobacterium PCC 7702
Mastigocladopsis repens PCC 10914
Calothrix sp. PCC 7103
Calothrix sp. PCC 6303
Nostoc sp. PCC 7524
Nostoc sp. PCC 7120
Anabaena variabilis ATCC 29413
Nostoc sp. PCC 7107
Cylindrospermum stagnale PCC 7417
Anabaena cylindrica PCC 7122
Anabaena sp. PCC 7108
Raphidiopsis brookii D9
Cylindrospermopsis raciborskii CS-505
Nostoc azollae 0708
Nodularia spumigena CCY 9414
Calothrix sp. PCC 7507
Microchaete sp. PCC 7126
Nostoc punctiforme PCC 73102
Tolypothrix sp. PCC 9009
Rivularia sp. PCC 7116
Synechocystis sp. PCC 7509
Gloeocapsa sp. PCC 7428
Chroococcidiopsis thermalis PCC 7203
Dactylococcopsis salina PCC 8305
Halothece sp. PCC 7418
Spirulina subsalsa PCC 9445
Spirulina major PCC 6313
Unidentified cyanobacterium UCYN-A
Cyanothece sp. CCY 0110
Cyanothece sp. ATCC 51472
Cyanothece sp. ATCC 51142
Crocosphaera watsonii WH 0003
Crocosphaera watsonii WH 8501
Cyanothece sp. PCC 8802
Cyanothece sp. PCC 8801
Synechocystis sp. PCC 6803
Pleurocapsa sp. PCC 7327
Microcystis aeruginosa NIES-843
Microcystis aeruginosa PCC 7806
Cyanothece sp. PCC 7822
Cyanothece sp. PCC 7424
Prochloron didemni P1
Gloeocapsa sp. PCC 73106
Synechococcus sp. PCC 7002
Leptolyngbya sp. PCC 7376
Cyanobacterium stanieri PCC 7202T
Geminocystis herdmanii PCC 6308T
Cyanobacterium aponimum PCC 10605T
Stanieria cyanosphaera PCC 7437
Pleurocapsa sp. PCC 7319
Xenococcus sp. PCC 7305
Chroococcidiopsis sp. PCC 6712
Moorea producta 3LT
Coleofasciculus chthonoplastes PCC 7420
Microcoleus sp. PCC 7113
Crinalium epipsammum PCC 9333
Chamaesiphon minutus PCC 6605
Geitlerinema sp. PCC 7105
Synechococcus sp. RCC307
Prochlorococcus marinus NATL 2A
Prochlorococcus marinus NATL 1A
Prochlorococcus marinus, subsp. pastoris CCMP 1986
Prochlorococcus marinus MIT 9515
Prochlorococcus marinus AS 9601
Prochlorococcus marinus MIT 9301
Prochlorococcus marinus MIT 9215
Prochlorococcus marinus MIT 9202
Prochlorococcus marinus MIT 9312
Prochlorococcus marinus MIT 9211
Prochlorococcus marinus, subsp. marinus CCMP 1375T
Prochlorococcus marinus MIT 9313
Prochlorococcus marinus MIT 9303
Synechococcus sp. WH 8102
Synechococcus sp. CC 9605
Synechococcus sp. WH 8109
Synechococcus sp. BL 107
Synechococcus sp. CC 9902
Synechococcus sp. WH 7805
Synechococcus sp. WH 7803
Synechococcus sp. CC 9311
Synechococcus sp. WH 8016
Synechococcus sp. RS 9916
Synechococcus sp. RS 9917
Synechococcus sp. WH 5701
Synechococcus sp. CB 0205
Synechococcus sp. CB 0101
Cyanobium gracile PCC 6307T
Cyanobium sp. PCC 7001
Synechococcus elongatus PCC 7942
Synechococcus elongatus PCC 6301
Prochlorothrix hollandica PCC 9006T
Leptolyngbya sp. PCC 6406
Nodosilinea nodulosa PCC 7104
Synechococcus sp. PCC 7335
Leptolyngbya sp. PCC 7375
Geitlerinema sp. PCC 7407
Leptolyngbya boryana PCC 6306
Thermosynechococcus elongatus BP-1
Synechococcus sp. PCC 6312
Cyanothece sp. PCC 7425
Acaryochloris marina MBIC11017T
Acaryochloris sp. CCMEE 5410
Synechococcus sp. PCC 7502
Pseudanabaena sp. PCC 6802
Pseudanabaena sp. PCC 7429
Pseudanabaena sp. PCC 7367
Synechococcus sp. JA-3-3Ab
Synechococcus sp. JA-2-3B
Synechococcus sp. PCC 7336
Gloeobacter violaceus PCC 7421T
0.3

References

Shih, P.M., Wu, D., Latifi, A., Axen, S.D., Fewer, D.P., Talla, E., Calteau, A., Cai, F., Tandeau de Marsac, N., Rippka, R., Herdman, M., Sivonen, K., Coursin, T., Laurent, T., Goodwin, L., Nolan, M., Davenport, K.W., Han, C.S., Rubin, E.M., Eisen, J.A., Woyke, T., Gugger, M., and Kerfeld, C.A. (2013). Improving the coverage of the cyanobacterial phylum using diversity-driven genome sequencing. *Proc Natl Acad Sci U S A* 110, 1053-1058. doi: 10.1073/pnas.1217107110.
